# Choosing and accessing COVID-19 treatment options: a qualitative study with patients, caregivers, and health care providers in Lebanon

**DOI:** 10.1186/s12961-024-01131-9

**Published:** 2024-03-27

**Authors:** Reem Hoteit, Aya Hassoun, Elie Bou Sanayeh, Marie Christelle Saade, Gladys Honein-AbouHaidar, Elie A. Akl

**Affiliations:** 1 Clinical Research Institute, American University of Beirut, Riad-El-Solh, P.O. Box: 11-0236, Beirut, 1107 2020 Lebanon; 2 School of Public Health, University of Saskatchewan, Saskatoon, SK S7N 5A2 Canada; 3 Department of Internal Medicine, American University of Beirut Medical Center, Beirut, Lebanon; 4 Rafic Hariri School of Nursing, American University of Beirut, Beirut, Lebanon; 5 Department of Health Research Methods, Evidence, and Impact (HEI), McMaster University, Hamilton, Canada

**Keywords:** COVID-19, Medication accessibility, Medication shortages, Treatment decisions, Economic crisis, Black market, Healthcare system, Healthcare professionals

## Abstract

**Background:**

The coronavirus disease 2019 (COVID-19) pandemic has strained healthcare systems globally, particularly in terms of access to medicines. Lebanon has been greatly affected by the pandemic, having faced concomitant financial and economic crises. The objective of the study was to understand the experiences of patients with COVID-19 in Lebanon, as well as those of their families, and healthcare providers, with regards to their treatment decisions and accessibility to COVID-19 medicines.

**Methods:**

For this qualitative study, we conducted 28 semi-structured interviews. We used purposive sampling to recruit participants with a diverse range of perspectives. The data collection phase spanned from August to November 2021 and was conducted virtually. After transcribing and translating the interviews, we employed thematic analysis to identify recurring themes and patterns.

**Results:**

In total, 28 individuals participated in this study. Participants highlighted challenges owing to the COVID-19 pandemic and economic crisis. Accessing COVID-19 medicines posed major hurdles for physicians and patients, given limited availability, global shortages, local circumstances, community hoarding and stockpiling by pharmacies. Providers based treatment decisions on research, local and international practice guidelines, experiences and expert feedback. Patients sought information from social media, community members and physicians, as well as through word of mouth. Accessing medicines involved navigating the healthcare system, the black market, charities, personal networks and political parties and sourcing from abroad. The medicines were either free, subsidized or at inflated costs.

**Conclusions:**

This study highlights the diversity and complexity of factors influencing decision-making and accessing medicines during the COVID-19 pandemic in Lebanon. Future research should explore strategies for ensuring medicine access during crises, drawing insights from comparative studies across different countries.

## Introduction

The emergence of the coronavirus disease 2019 (COVID-19) instigated a global health crisis, presenting formidable challenges to healthcare systems and economies across the world [1, 2]. Since its first appearance in December 2019 in China, severe acute respiratory coronavirus 2 (SARS-CoV-2) has infected around 700 million individuals, resulting in a staggering death toll exceeding 6.9 million by November 2023 [3, 4]. Owing to COVID-19-related lockdowns and the heightened demand for essential medications, drug shortages have become a significant global problem [5, 6].

Prior to the pandemic, healthcare systems in low- and middle-income countries (LMICs) suffered from limited financial resources, healthcare workforce shortages and unavailability of medications [7–9]. The pandemic further strained these already fragile health systems [2]. For example, the strong demand for medications to treat COVID-19 patients, including analgesics, sedatives, antibiotics, hydroxychloroquine and remdesivir, considerably affected medication accessibility and inadvertently encouraged black market activity [10–13]. Escalating medication prices rendered these crucial medications unaffordable for many patients, particularly in LMICs [13–15].

The economic and financial crises in Lebanon, coupled with the Beirut Port’s destruction, severely impacted the entire healthcare sector, affecting hospitals, healthcare providers and the pharmaceutical and medical supply industry [16]. The COVID-19 pandemic worsened this situation, posing two major challenges: the selection of appropriate therapies and ensuring access to these treatments [17, 18]. This shortage of prescription drugs in Lebanon peaked during the COVID-19 pandemic [19].

Factors influencing decisions regarding COVID-19 treatment can be complicated and multidimensional. They encompass an individual patient’s medical history, comorbidities and risk factors, as well as the availability and efficacy of various therapeutic options [20]. Therapeutic management in the early stages of the pandemic was challenging owing to uncertainty and continuously evolving evidence [21]. Clinicians attempted to manage COVID-19 using a variety of treatments that targeted numerous possible mechanisms, such as antiviral, anti-inflammatory and immunomodulatory drugs [22]. There was also misinformation in various media outlets about the benefits of some medications for either preventing or treating COVID-19 [23]. This resulted in an increase in risky self-medication with several over-the-counter medications [2, 24].

The objective of the study is to understand the experiences of patients with COVID-19 in Lebanon, as well as those of their families, and healthcare providers, with regards to their treatment decisions and accessibility to COVID-19 medicines.

## Methods

### Study design

This study adopted a descriptive qualitative research design using semi-structured individual interviews (refer to Appendix 1 for the interview guide). The qualitative approach utilized is rooted in naturalistic inquiry and offers a wide array of theoretical or philosophical orientations, sampling techniques and data-gathering strategies [25].

### Participants

We recruited participants from different regions in Lebanon. Eligible participants belonged to one of the following groups:

1. physicians and nurses directly involved in caring for patients diagnosed with COVID-19
2. hospital and community pharmacists involved in dispensing medications for patients diagnosed with COVID-19
3. patients previously diagnosed with COVID-19
4. family members or caregivers of patients previously diagnosed with COVID-19.

We excluded patients who were psychologically unable to participate or provide coherent and clear descriptions of their experiences.

### Sampling and recruitment

We used purposeful sampling by approaching individuals belonging to the groups of interest. We also used snowballing sampling by asking participants to refer us to other eligible individuals. Additionally, physicians and pharmacists assisted in the recruitment of potential former patients and caregivers. The Institutional Review Board (IRB) at the American University of Beirut (AUB) approved the study. All participants provided oral consent prior to participation. The interviewers took all precautions to guarantee participants’ anonymity and confidentiality. Participants were informed that their participation was entirely voluntary and that they could opt-out at any time.

### Data collection

Following an explanation of the study’s objectives, we interviewed participants virtually in either English or Arabic, depending on their preferences. We audio-recorded interviews following participants’ consent. We conducted a total of 28 interviews, and we ceased to collect data when thematic saturation was reached, that is, no new themes emerged from the data analysis [26].

Two team members (AH and EBS) conducted the interviews between August and November of 2021. The individuals received thorough training on conducting interviews, focusing on techniques to remain neutral and nonjudgemental and to sustain the interviewees’ engagement in the subject matter. To enhance the quality of data collection, we held regular debriefing meetings following the initial interviews. These meetings provided an opportunity for reflection on the data collection process and identification of areas of improvement.

### Data analysis

The interviewers transcribed the audio-recorded interviews, and translated them into English when applicable. Another team member (RH) verified transcript accuracy by checking them against the audio recordings. We employed Quirkos, a qualitative analysis software, for coding and organizing the data. We applied Braun and Clarke’s six-step thematic analysis approach [27]. In phase 1, GHA and RH read a few transcripts independently to familiarize themselves with the information and established a preliminary framework for data coding. In phase 2, they independently annotated the transcripts line by line. They assigned labels to each idea (coding), leaving room for new codes as they emerged. In phase 3, GHA, EAA and RH reviewed the coded transcripts and identified emerging themes, along with quotes that illustrated each theme. In phase 4, GHA, EAA and RH reviewed and refined the list of emerging themes, and created a thematic map. In phase 5, they outlined the final thematic framework. Finally, in phase 6, we developed a complete narrative of the findings and selected interviewee quotes for each theme and sub-theme.

### Increasing rigour

All interviewers received training in interviewing skills, maintaining consistency and rigour [28]. We also made sure that interviewers had no prior relationship with participants, fostering objectivity and minimizing bias [28]. We interviewed participants in their preferred language as a way to ensure their understanding of the questions and their ability to easily express their thoughts [28, 29]. To ensure transferability, we employed triangulation by compiling viewpoints of various population groups [29]. We halted data collection upon reaching saturation [30], ensuring comprehensive data coverage and depth. We verified transcript accuracy by checking them against the audio recordings [31]. Three members of our research team (GHA, EAA and RH) actively participated in the analysis and the generation of codes, themes and subthemes. In reporting this study, we adhered to the highest standards by following the Consolidated Criteria for Reporting Qualitative Research (COREQ) checklist [32].

## Results

### Demographics

We recruited 28 participants: 3 community pharmacists, 4 hospital pharmacists, 8 physicians, 1 nurse, 3 patients and 9 caregivers. The interviews lasted about 40 min on average.

### Emerging themes

**Fig. 1:**
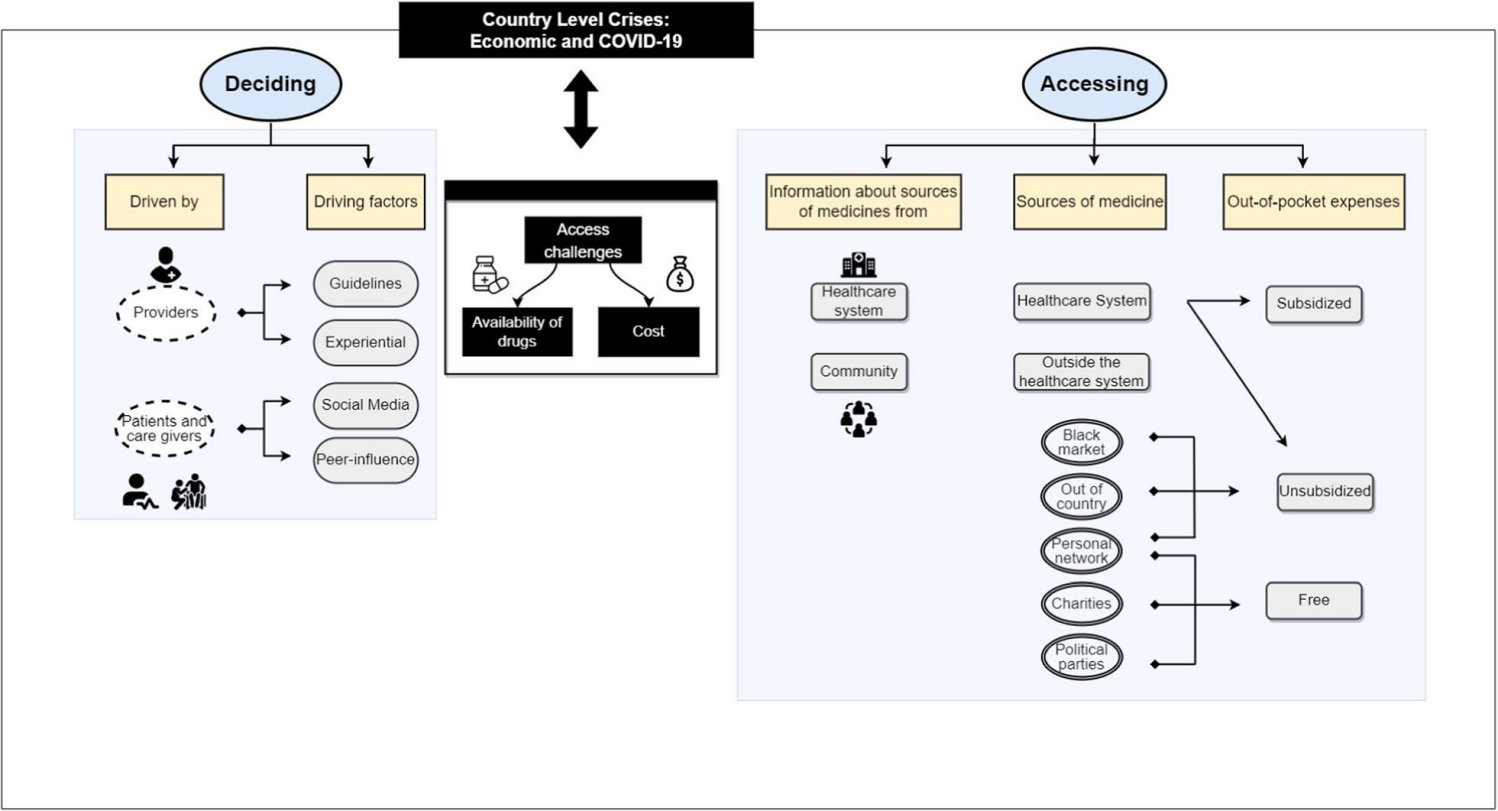
Factors influencing decision-making and accessing medicines during the COVID-19 pandemic in Lebanon

The following themes emerged in relation to the experiences of participants with regards to treatment decisions and accessibility to COVID-19 medicines: country crises, access challenges, cost challenges, drivers for providers’ decision-making, drivers for patients and caregivers’ decision-making and accessing medicines (Fig. 1).

When the majority of participants expressed the same opinions, we used the term “most participants”; otherwise, we used the terms “many” or “few” as appropriate. When citing quotes from participants, we used the following acronyms: physicians (Phys), nurses (Nurse), community pharmacists (C-Pharm), hospital pharmacists (H-Pharm), patients (Pt) and caregivers (Cg).

#### Country crises

Most participants discussed current events in the country, including the COVID-19 pandemic, the economic crisis and the financial crisis. “Because of the current situation in Lebanon, we’re seeing things we never thought we would” (H-Pharm 02). “COVID-19 came around in March 2020, and Lebanon had already started its economic crisis” (H-Pharm 04).

Participants noted their experiences with the financial crisis and the closure of banks. “I woke up in the morning at 6:45 am, wore my clothes and went down to the bank but found it closed […], I went down to [..], same thing, it closed as I was on my way so I said, ‘where should I get them the money now?; they asked for money for the tests” (Cg 08).

One caregiver also mentioned that the economic crisis had forced them to work multiple jobs. “I work two jobs but now if you work in Lebanon the salaries are not enough” (Cg 08).

In addition to this, participants complained about how the country’s financial situation impacted access to medicines. “The purchasing power of the patients had already decreased. And like all countries when it comes to medication and the healthcare system, which was already collapsing in Lebanon, it’s common that in the end, the patient has to self-fund their treatment. And patients in low economical standing will have no access to treatment” (H-Pharm 04).

#### Access challenges

The crisis impacted the supply of COVID-19 medications. First, there were drug shortages directly related to the COVID-19 medicines, similar to the global crisis “… we saw that everyone is facing this, worldwide… Then we went into shortages, we didn’t have anymore because the consumption had increased” (H-Pharm 02). “Even the Colchicine, we heard about it, and we went around and looked for it and it was very hard for us to find it easily” (Cg 02).

“For example, remdesivir they used for my mother-in-law we got six injections, and the first day we tried to get it we called the pharmacies they told us they don’t have it. In the hospital, there was no remdesivir because it was getting brought based on an order from the company and it needed a prescription from the doctor to obtain” (Cg 01).

Second, the challenging circumstances in Lebanon had an additional impact on the accessibility of medicines. “The effect of the medications is unrelated to COVID-19. I mean, the availability of the medications. Now, even after COVID-19 has decreased, we have shortages in medications that are unrelated to COVID-19, it has to do with the economic situation. So, it’s not COVID-19 that made the crisis in medications, not at all” (Phys 08). “It was hard to provide medications because of the terrible situation of Lebanon” (Nurse 01).

Many pharmacists discussed community hoarding. “People were running to the pharmacies to secure one of these medications even if they did not need it at the time, just for the sake of keeping it at home just in case, which led to a huge shortage of supply” (C-Pharm 02).

However, patients were concerned about pharmacies stockpiling medications. “Exactly, because of the economic crisis selling the drug was not beneficial for the pharmacies so they started keeping it for emergency cases and selling it at the black market rate. After all the demand was very high” (Cg 07).

Few physicians mentioned lack of availability of medicines specifically in the hospitals, particularly for the new medications. “They had to get the medications outside of the hospital…the shortage was due to the fact that basically, this is a new medication, and it hasn’t been brought to Lebanon yet.” (Phys 08).

A few pharmacists stated that they always had a backup plan. “To be honest we never had really bad shortages, we never fully ran out, we always had a plan B. When there was no more dexamethasone for IV, we prepared other corticoids, even if it wasn’t mentioned in any studies or guidelines. We used to prepare them and keep them as backups in case they were ever needed” (H-Pharm 04).

#### Cost challenges

Both providers and patients noted unaffordable costs as another factor affecting access to medications, considering the devaluation of the currency. “The single pill got to about 50 USD, it was very expensive. So, it was really expensive for most people. Its actual price was 5000 Lira” [H-Pharm 04; note that at the time 5000 Lira was worth less than 5 US dollars (USD)]. “Even tablets like vitamins are available but they are very expensive, not everyone can afford them” (Cg 04).

Patients complained about price manipulation. “The prices were definitely manipulated because when I would buy a medication I would find more than one price tag… The lozenges for her throat used to cost 19 000 Lira and now it costs 45 000 Lira. They put more than one label on the box of medication, there are about three price tags on it” (Cg 06).

Additionally, it was mentioned that the pricing of medications was changed to the US currency. “Yes, most of the time they were fresh dollar” (Phys 08).

#### Drivers of decision-making for providers

During the COVID-19 pandemic, several factors influenced decisions by providers, patients and caregivers about which medicines to use or not use.

Owing to the rapid development of evidence, providers were compelled to rely on research to prescribe certain medications. “There was a committee that used to review the data available and to review all the evidence at the time and make the decisions.” (Phys 04). “… the COVID-19 protocol changed every couple of days. Every once in a while, a new study would appear, a new update, and it would change again” (H-Pharm 01).

Providers also relied on clinical practice guidelines developed either locally or internationally, for example, by the WHO. “The medications we were prescribing were based on WHO” (Phys 03). Although they relied on those guidelines, some providers expressed hesitations about them. “Yes, we were following the guidelines of treatment of COVID-19, we would tell this is the medication that needs to be taken because this is what the guidelines say. We are not sure of the guidelines, but this is what is needed now” (Phys 09).

Additionally, the providers’ prior experience or trial and error played a role in the decision-making process. “At first, personally, I did not have much experience with this disease but later on and after I acquired some experience, I was finally able to give my own opinion on the matter” (C-Pharm 01).

Reliance on local peers with different specialities played a significant role in decision-making. “Because we had several specialities – cardiovascular, internal medicine, and others – everyone did their research and every week we would meet and explain to each other… Everyone gave their inputs and propositions, in their own specialities, about which drugs might be good, and which drugs were used in which cases” (H-Pharm 04).

International expertise was also sought during the pandemic. “We also had video conferences with hospitals and ICUs in France and the United States, because we had physicians that went and studied in those countries and still had contacts, we did one video conference with France, and one with the US to ask about their protocols. And there were discussions about what’s best. And when Actemra was first being used by the ones we talked to somewhere in Houston, we weren’t using it yet in our hospital. After the video conference, they found that their patients are showing good results, so it was added to our protocol..” (H-Pharm 04).

It is of note that the country’s situation and drug availability influenced the decision-making process. As expressed by many doctors: “We were following the new guidelines, taking into consideration the situation of the country and the availability of the drugs and imaging” (Phys 02).

#### Drivers of decision-making for patients and caregivers

Patients and caregivers relied on social media to decide on drug purchases. “Yes. Honestly, they saw me crying and I had posted on Instagram that if anyone please could help with their experience because there were no studies at that time” (Cg 03). “At first people used to wait for what the media says and then come running to the pharmacies to buy these medications, it happened first with vitamin C then 2 weeks later with vitamin D then it was the zinc 25 mg and then zinc 50 mg turns” (C-Pharm 01). “Even the colchicine, we heard about it on social media, and we went around and looked for it and it was very hard for us to find easily” (Cg 02).

Patients and caregivers were also influenced by people in their communities, some of whom had experience with COVID-19. “Other people around us who also had corona, everyone that got corona would say take this and do this” (Pt 02). “My dad caught it in the beginning, so I started asking people to see what we could do. One of my friends told me that there was a person who took this medicine, and they told me to try it, so I decided to do that” (Cg 03).

Patients reported different attitudes about consulting with their doctors. “We heard about remdesivir and asked the doctor, he told us he can’t advise us to take it or not, if we would like to try it based on other patients and not on medical research then go ahead” (Cg 07). Some made decisions on their own on the basis of word of mouth. “My friend called me and told to me not listen to the doctors and to take zithromax. I bought it and took one pill” (Pt 08).

#### Accessing medicines

We have identified two subthemes under the theme of “accessing medicines”: information about how to get the medicines and the sources of medicines.

### Information about how to get the medicines

Typically, patients obtained information about how to get the medicines from healthcare providers, including nurses, physicians and pharmacists. “Yes sure!… [local charities] used to give those medications (remdesivir, Actemra, etc.) for free. And there were some other providers. We used to indicate the providers to the families of the patients” (Nurse 01).

Patients also inquired about the source of medicines from recovered COVID-19 patients: “From other people around us who also had corona” (Pt 02).

### Sources of medicines

Patients and their caregivers obtained the medicines either through the healthcare system or from outside the healthcare systems, including the black market, nongovernmental organizations (NGOs), personal networks, political parties and outside of the country.

#### Healthcare system

When patients were admitted, few reported that COVID-19 medicines were available in the hospital. “They were all found in the hospital” (Cg 05). However, for several patients, their family members had to seek medicines from community pharmacies. “We got them from the pharmacy” (Cg 01).

Medicines were obtained at no cost thanks to a subsidy by the Lebanese government. “So, it was for free if it was from the Ministry” (Cg 02). However, some other medicines were purchased on an unsubsidized basis and at high cost. “A few pills were for 1 300 000 Lebanese Lira in the pharmacy” (Cg 02).

#### Black market

Owing to the limited supply and urgent need for COVID-19 medications, the black market flourished. “There were two more weeks, and the Ministry was supposed to secure it, but we needed it urgently, so they gave us the number of someone who sells it in the black market and he got it for us” (Pt 03).

The black market was viewed as a double-edged sword because it allowed access but at an inflated cost. “They gave us five remdesivir and one Actemra for US$ 1200” (Cg 07). “The remdesivir is like. So, he made us pay US$ 700 for one,.. So, US$ 4200 for six pills” (Pt 03).

Because of the country’s financial crisis, inflated black market prices presented a major challenge for patients. “… it was a challenge for us to financially secure the medicine. And of course, him asking for US$ 4200 cash was not something easy for someone to get and pay, but if it is the only solution of course we would do it” (Pt 03).

#### Charities

Charities supported patients in accessing their medicines either for free or through financial support. “Suppose I were to get COVID-19 now, my name would go down at the municipality and they get you vitamin C and vitamin D – a charity organization, not from the government” (Cg 08). “I paid 1 million and the rest was on the charity organization” (Cg 08); “For ivermectin there were a lot of organizations trying to supply it, it’s a very cheap drug… that costs US$ 4. There were also a lot of organizations trying to supply remdesivir, ‘Hariri’ (a local charity) was trying to help with it since hospitals did not have it, people were going to her villa to get it, it costs I think about US$ 4000” (Phys 02).

#### Personal network

Caregivers of patients with COVID-19 used their personal networks, including family and friends: “Also, from a person who knows a pharmacist he’s friends with, they got them for us” (Cg 02). “We had to get the baricitinib from someone we know, who got it for us from the Ministry” (Cg 02).

#### Political parties

Political parties also supplied medicines to their supporters. “There were parties that were obtaining them, like [name of political parties]. Those were for free as a donation from [name of political party]” (Pt 02).

#### From outside of the country

Typically, family or friends helped by purchasing medicines while travelling. “At the time, an Iraqi who is friends with my relative got it and he paid US$ 400” (Pt 08).

Out-of-country purchases were driven by either lack of local supply or inflated costs. “The ivermectin was still not in Lebanon, so we got it elsewhere, from a woman who lives in Africa, she got it for us and sent it. And we started with cortisone, this is from day 1” (Cg 02). “My cousin sent it from Sweden, she sent zinc and vitamin C because vitamin C here now costs 60 000 Lira, before it cost 14 000 Lira and now it costs 60 000” (Cg 06).

## Discussion

This study aimed to to understand the experiences of patients with COVID-19 in Lebanon, as well as those of their families, physicians, nurses and pharmacists, with regards to their treatment decisions and accessibility to COVID-19 medicines.

The participants highlighted the country’s difficulties, especially the severe impact of COVID-19 pandemic and the economic crisis. Access to COVID-19 medicines and their costs were major challenges according to the three groups interviewed. Limited access related to global shortage of medicines, the local challenging circumstances, community hoarding (according to pharmacists) and stockpiling by pharmacies (according to patients). For providers, the decision-making process for COVID-19 treatments was shaped by research evidence, local and international practice guidelines, previous experiences and feedback from both local and international experts. Patients and their caregivers relied on social media, community members, physicians and word of mouth. Information on how to get the medicines was obtained from either healthcare providers or patients who recovered from COVID-19. Accessing medicines involved navigating through the healthcare system (hospitals and pharmacies), as well as outside that system, including the black market, charities, personal networks, political parties and outside of the country. Across these different sources, the medicines were either free, subsidized or at inflated costs.

### Comparison to similar studies

A major finding in our study was the accessibility of patients and healthcare providers to needed medicines. This is corroborated by other studies conducted in Lebanon [33, 34] and low-and middle-income countries [35]. The global impact of lockdowns on medicine manufacturing, supply and distribution contributed to shortages during the high-demand period of the COVID-19 pandemic [36, 37]. Furthermore, Lebanon has faced severe economic and financial crises starting in 2019, which severely hindered the capacity to import vital healthcare equipment and medicines [38, 39]. Indeed, the World Bank characterized the crisis as “among the world’s worst since the 1850s” [40]. The lack of government reimbursement further hindered hospitals in procuring necessary medications and medical supplies [41]. Consequently, individuals affected by COVID-19 in Lebanon resorted to unregulated sources, including the black market, often resulting in inflated prices and the risk of expired or counterfeit drugs [13–15, 42].

Moreover, in line with our findings, other studies found that healthcare providers followed both international and national guidelines when deciding on potential treatments for COVID-19 patients [43, 44]. However, in the absence of effective medications, discussion on various social media platforms encouraged self-medication and the use of herbal medicines [45, 46]. In addition, a recent study conducted in Jordan assessing the usage of medications and natural products amidst the second wave of COVID-19 revealed that individuals primarily sought guidance from family and friends, with social media platforms serving as significant sources of advice concerning the use of these medications [47]. The same study showed that pharmacists notably played a significant role in guiding individuals on choosing these treatments compared with other healthcare providers [47].This highlights the impact of social media on treatment choices and emphasizes the need for disseminating accurate and evidence-based information.

### Strengths and limitations

To our knowledge, this is the first study in Lebanon to comprehensively explore the interplay between country crises and medication accessibility during the COVID-19 pandemic, offering valuable insights into the unique challenges faced by the country. We explored in-depth the lived experiences of our participants, ensuring the representation of the perspectives of healthcare providers, patients and caregivers. Also, we used a rigorous qualitative methodology (please refer to the “Increasing rigour” section).

There are several limitations to consider. Firstly, the study focuses primarily on Lebanon, which may limit the findings’ generalizability to other countries with distinct settings and healthcare systems. Moreover, there is a possibility of recall bias among participants, as their recollections of events and experiences concerning medication accessibility during the crisis might be influenced by subjective interpretations or memory lapses. Additionally, the sampling technique employed might introduce selection bias, as participants were recruited through purposive sampling. Furthermore, it is important to note that this study is based on a specific snapshot in time during the COVID-19 pandemic. Consequently, its findings may not fully encapsulate the dynamic and evolving nature of the crisis or account for potential shifts in medication accessibility and decision-making processes over time.

## Conclusions

This study sheds light on the wide range of factors influencing treatment decisions during the COVID-19 pandemic in Lebanon. It also unveils how patients and their families had to access medications either through the formal healthcare systems or through black markets and other channels. Plans are needed to address medicine availability, affordability and equitable distribution during similar future crises. There is an urgent need for collaborative efforts involving stakeholders, policy-makers and key systems such as Meditrack and AMAN within the Ministry of Public Health [48, 49]. These initiatives are intended to establish resilient and sustainable drug supply chains and to ensure timely and equitable access to medications for all individuals, particularly in times of crisis. Furthermore, improving collaboration among healthcare providers, expediting medication access and creating patient support programs can alleviate the difficulties that people seeking treatment confront. For example, streamlining communication between hospitals, pharmacies and primary care doctors could speed up the prescription and dispensing processes.

Future research should focus on effective strategies to ensure medicine access during crises. Comparative research across different countries can provide valuable insights into successful tactics that can be tailored across different countries.

## What is already known on this topic

- global healthcare systems have been strained owing to the COVID-19 pandemic, leading to challenges in medicine access; and
- Lebanon’s healthcare system has been significantly impacted by the pandemic and financial crises, affecting the availability of medicines.

## What this study adds

- it uncovers key factors influencing both healthcare providers and patients in their treatment decisions, providing a comprehensive perspective; and
- it describes varied sources for medicines, including informal networks and the black market.

## How this study might affect research, practice or policy

the findings emphasize the necessity for strategies that ensure continuous medicine access, particularly during times of crises and economic instability.

## Data Availability

The datasets analysed during the current study available from the corresponding author on reasonable request.

## Abbreviations

AUB: American University of Beirut
Cg: Caregivers
COREQ: Consolidated Criteria for Reporting Qualitative Research
COVID-19: Coronavirus disease 2019
C-Pharm: Community pharmacists
H-Pharm: Hospital pharmacists
IRB: Institutional review board
LMICs: Low- and middle-income countries
MOPH: Lebanese Ministry of Public Health
NGOs: Nongovernmental organizations
Phys: Physicians
Pt: Patients
SARS-CoV-2: Severe acute respiratory syndrome coronavirus 2
USD: US dollar
